# Smartphone-Enabled Colorimetry

**DOI:** 10.3390/s23125559

**Published:** 2023-06-14

**Authors:** Leonardo Ciaccheri, Barbara Adinolfi, Andrea Azelio Mencaglia, Anna Grazia Mignani

**Affiliations:** CNR—Istituto di Fisica Applicata “Nello Carrara”, 50019 Sesto Fiorentino, FI, Italy; b.adinolfi@ifac.cnr.it (B.A.); a.mencaglia@ifac.cnr.it (A.A.M.); a.g.mignani@ifac.cnr.it (A.G.M.)

**Keywords:** smartphone, colorimetry, spectroscopy

## Abstract

A smartphone is used as a colorimeter. The performance characterization for colorimetry is presented using both the built-in camera and a clip-on dispersive grating. Certified colorimetric samples provided by Labsphere^®^ are considered as test samples. Color measurements directly performed utilizing the smartphone camera only are obtained using the RGB Detector app, downloaded from the Google Play Store. More precise measurements are achieved using the commercially available GoSpectro grating and related app. In both cases, to quantify the reliability and sensitivity of smartphone-based color measurements, the CIELab color difference Δ*E* between the certified and smartphone-measured colors is calculated and is reported in this paper. In addition, as an example of a practical application of interest for the textile industry, several samples of cloth fabrics with a palette of the most common colors are measured, and the comparison with the certified color values is presented.

## 1. Introduction

### 1.1. Why Use a Smartphone for Colorimetry?

The ubiquitous and exponentially increasing use of smartphones has inspired a wide community of scientists to use them as platforms for analytics based on colorimetry. In fact, there are several advantages offered by a smartphone-enabled colorimeter, such as: (i) portability—smartphones are small, lightweight portable devices that can be easily carried around for on-the-go fieldwork or outdoor measurements; (ii) cost-effectiveness—that makes colorimetry accessible to a wider range of users who may not have the budget for specialized equipment; (iii) a high-quality camera that can capture images with high resolution and a good color accuracy—this makes them suitable for capturing and analyzing color values, particularly for applications where a high level of detail is required; (iv) easy to use: smartphones are user-friendly, and most people already know how to operate them; (v) integration with other features such as internet connectivity, GPS, and voice recording—these options can be easily integrated with colorimetric measurements, for example, to automatically save location data with each measurement, or to record voice notes or images alongside the results.

Indeed, the most important feature of the smartphone for colorimetry is that it has a built-in white LED light and a digital camera. Hence, by simply shining the LED light on a sample and detecting the reflected light, it is possible to achieve a good colorimetric assessment. More precise colorimetric measurements can be achieved by adding in front of the camera a dispersive grating while using the smartphone camera as a detector.

Recently, several reviews and their references showed the state of the art of smartphone-based colorimeters used as a general spectrophotometer for analytical chemistry and biochemistry [1,2,3,4,5,6], or modified for the readout of biosensors either for healthcare [7,8,9], including paper-based transducers [10], microfluidic devices for point-of-care applications [11,12,13], immunosensors for personalized medicine [14], and for food quality and safety monitoring [15,16], for the detection of hazardous substances [17], and for environmental applications [18,19].

### 1.2. Scope of This Paper

While colorimetry by smartphones has its benefits, it is important to note that the accuracy of measurements may not be as high as that of specialized equipment. The scope of this paper is to assess to what extent the colorimetric performance of a smartphone can be improved by using a clip-on dispersive grating. For this purpose, we selected as test samples certified color tiles, and we characterized the smartphone colorimeter in two cases:

1. using the built-in white LED and camera as a source and detector, respectively, and
2. using a dispersive grating clipped onto the camera for more precise measurements.

In both cases, the colorimetric coordinates obtained by the smartphone are compared with the certified values to evaluate the measurement precision and the color resolution. Additionally, as a practical example which is of interest for quality control in the textile industry, some cloth fabrics with the most common colors are measured, and the comparison with the previously certified color coordinates are given.

## 2. Materials and Methods

### 2.1. Samples for Color Measurements

Eight colorimetric tiles certified by Labsphere^®^ were considered as test samples, with the following colors: violet, purple, blue, cyan, green, yellow, orange, and red. Moreover, twelve samples of cloth fabrics with a palette of the most common colors were considered as test samples for a straightforward application in the textile industry. In fact, to optimize the use of pigments and their mixture, the textile industry frequently needs a quick color check during the dyeing process. In addition, fashion stylists like to communicate to the dying industry the colors they occasionally see, and a smartphone grabbing of the selected color then converted to chromatic coordinates by an app is a quick and effective method.

**Figure 1:**
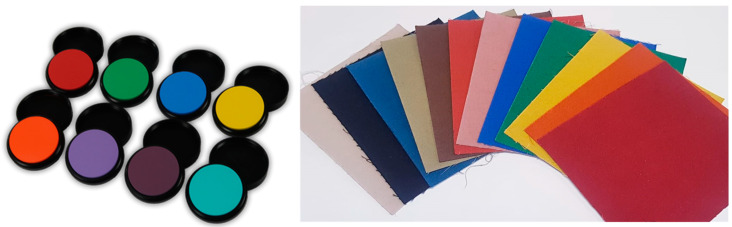
Test samples: LabSphere^®^ color standards (**left**), and cloth fabrics (**right**).

All measured tiles and cloth fabrics are shown in Figure 1 while Table 1 mentions the short codes we used for their identification. A Spectralon tile was used as a reference for all color measurements. The certified spectra of tiles were available from Labsphere^®^, and the cloth fabrics were previously certified by means of conventional spectrophotometric measurements. In practice, the reference tristimulus coordinates *XYZ* were available for all samples so that it was possible to compare the smartphone-measured values with the certified ones.

### 2.2. Setups for Color Measurements

**Figure 2:**
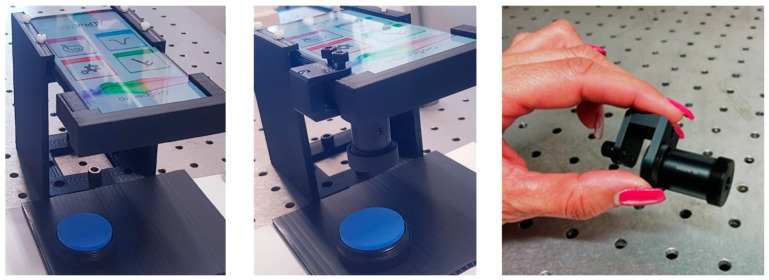
Setups for color measurements: using the smartphone alone (**left**), the clip-on grating (**center**), and the GoSpectro grating (**right**).

Figure 2 shows the setups used for reflection measurements, making use of the smartphone camera alone, or equipped by the commercially available clip-on GoSpectro grating [20]. This small component costs about EUR 400. The smartphone is a Huawei “P smart 2019” model, operated by Android 12. A 3D-printed plastic case is used to hold the smartphone with the same sample–smartphone distance during the color measurements, also allowing us to use the touch screen for operation.

### 2.3. Colorimetric Characterization

When the reflection measurements were carried out by using the smartphone camera alone, the red, green, and blue outputs of the video camera were converted into RGB coordinates by the RGB Detector app, downloaded from the Google Play Store. This app is supposed to have auto-calibrating capability, providing RGB coordinates independently of the model of camera used. They were then transformed into tristimulus *XYZ* coordinates by means of a conversion matrix shown in Equation (1) (conversion coefficients rounded up to the third decimal digit) [21].

Instead, for reflection measurements carried out using the GoSpectro grating, the column of CCD array on which the light was focused determined the wavelength. The wavelength scale was calibrated by using the spectral lines of a fluorescent lamp, and the red, green, and blue outputs of the whole CCD column were summed up to obtain the corresponding intensity. The *XYZ* tristimulus coordinates were obtained from the reflectance spectra by Equation (2).

(1) $$\left[\begin{array}{c} X\\ Y\\ Z \end{array}\right]=\left[\begin{array}{ccc} 0.412 & 0.358 & 0.180\\ 0.213 & 0.715 & 0.072\\ 0.019 & 0.119 & 0.950 \end{array}\right]\;\left[\begin{array}{c} R\\ G\\ B \end{array}\right]$$

(2) $$X=K\;\int_{780}^{380}L\left(\lambda \right)\;x\left(\lambda \right)\;d\lambda \;Y=K\;\;\int_{780}^{380}L\left(\lambda \right)\;y\left(\lambda \right)\;d\lambda \;Z=K\;\int_{780}^{380}L\left(\lambda \right)\;z\left(\lambda \right)\;d\lambda$$

where *x*(*λ*), *y*(*λ*), and *z*(*λ*) are the CIE color functions for calculating the tristimulus coordinates, and *L*(*λ*) is the luminance spectrum given by Equation (3):

(3) $$L\left(\lambda \right)=T\left(\lambda \right)\;L_{D65}\left(\lambda \right)$$

being *T* the transmittance (reflectance) spectrum, and *L_D65_* the luminance spectrum of D65 standard illuminance. D65 illuminant is a theoretical standard illuminant spectrum used by the Commission Internationale de l’Éclairage (CIE), which corresponds roughly to the midday light in Western/Northern Europe under a clear sky. Real sources can only approximate to the D65 spectrum. This is the reason why reflectance spectra (which only depend on samples) are used for calculating tristimulus coordinates. The spectrum of D65 and the color functions can be downloaded from the CIE website [22]. The *K* constant is calculated so that *Y* = 100 for the white Spectralon reference. Because the transmittance spectrum is calculated as the ratio between sample and reference spectra, it is independent of camera characteristics.

For both setups, all measurements were performed five times and the average and standard deviations were calculated.

Finally, in both cases, by considering the average values of the measurements, the CIELab coordinates L* (clarity), a* (red–green balance), b* (blue–yellow balance) were calculated by formulas in Equation (4).

(4) $$L^{*}=116\;{\left(\frac{Y}{Y_{n}}\right)}^{1/3}-16$$

$$a^{*}=500\;\left[{\left(\frac{X}{X_{n}}\right)}^{1/3}-{\left(\frac{Y}{Y_{n}}\right)}^{1/3}\right]\;b^{*}=500\;\left[{\left(\frac{Y}{Y_{n}}\right)}^{1/3}-{\left(\frac{Z}{Z_{n}}\right)}^{1/3}\right]$$

Equation (4) gives the nonlinear transformation equation from the tristimulus to CIELab 1976 color space, in accordance with CIE directives [22]. The color differences ΔE were computed by Equations (5) and (6):

(5) $$\Delta E^{*}={\left(\Delta L^{*2}+\Delta a^{*2}+\Delta b^{*2}\right)}^{1/2}$$

(6) $$\Delta L^{*}=L_{2}^{*}-L_{1}^{*}\;{\Delta a}^{*}=a_{2}^{*}-a_{1}^{*}\;{\Delta b}^{*}=b_{2}^{*}-b_{1}^{*}$$

being $\left(L_{1}^{*},\;a_{1}^{*},\;b_{1}^{*}\right)$ and $\left(L_{2}^{*},\;a_{2}^{*},\;b_{2}^{*}\right)$ the CIELab vectors of the reference and measured colors, respectively. Additionally, the resolution of the color measurements, δ_E*_, was computed as Equation (7):

(7) $$\delta_{E^{*}}={\left(\;\delta_{L*}^{2}+\delta_{a*}^{2}+\delta_{b*}^{2}\;\right)}^{1/2}$$

where δ_L*_, δ_a*_, and δ_b*_ are three times the standard deviations of L*, a*, and b*.

## 3. Results and Discussion

**Figure 3:**
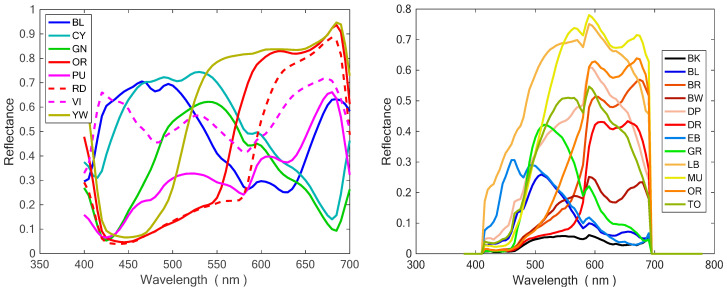
Spectra of tiles (**left**), and cloth fabrics (**right**) measured using the GoSpectro grating.

Figure 3 shows the spectra of tiles and cloth fabrics measured using the GoSpectro grating, and Table 2 summarizes the results achieved. As expected, the best results were obtained by using the grating. However, when we also used the smartphone camera alone, acceptable results were obtained, especially considering that simply using the smartphone gives us a very basic measurement, and that in many cases a quick color evaluation is more than sufficient. When we used the grating, yellow and cyan tiles showed the smallest color differences, while the biggest differences were for purple and red tiles.

The main reason why the smartphone camera alone provided worse results is that the RGB to *XYZ* conversion matrix requires the RGB measurement to be made by using a standard D65 illuminant, which is usually not the case. On the contrary, the measurements achieved by using the GoSpectro grating do not suffer from this problem because the reflectance spectrum does not depend on the light source. Multiplying this reflectance spectrum by the spectrum of the D65 illuminant, which can be downloaded from the CIE website (together with *XYZ* functions), we can calculate the correct tristimulus coordinates.

A source of error when using the GoSpectro grating comes from the fact that the 380–400 nm and 700–780 nm bands are not adequately covered by the CCD detectors. The pixels of the smartphone camera are coated by band–pass filters for the red, green, and blue bands. The transmittance of these filters drastically drops outside the 400–700 nm band, as it appears in the spectra of Figure 3. However, this should not be the main cause because the tristimulus functions have little weights in these bands. Another error could be caused by the white reference spectrum. In fact, if it does not have a truly flat spectrum, it could distort the reflectance spectrum. Moreover, an excessive illumination could bring some parts of the CCD array close to saturation, determining a nonlinear response. This problem affects both the GoSpectro and the RGB Detector apps. Further studies are currently ongoing on this point.

## 4. Conclusions

A smartphone was used for colorimetry, using the built-in camera only, and by means of a commercially available clip-on grating. Certified color standards and cloth fabrics were measured, and the color differences with respect to the certified values were presented.

Although measurements with the same precision as a spectrophotometer could not be achieved, surely noteworthy is the importance of having in the pocket an instrument that allows us to obtain a good colorimetric assessment in practice at no cost. Indeed, using a smartphone for colorimetry opens up new solutions for a wide class of problems in many industrial, environmental, and agricultural applications as well as for artisans and consumers who need to obtain quick information on the composition or nature of various materials: varnishes, dyes, bricolage, and many others.

In addition, because almost everyone has a smartphone in their pocket, colorimetric-based measurements enable new applications in citizen science. Indeed, the participation of the population in scientific experiments is expected to grow in the coming years, and simple tools are required by the community for data collection and volunteer mapping. A straightforward example could be the data collection for marine and environmental research addressed at the protection of the ecosystem by means of an integrated and synergic scientist–citizen action. Particularly for these applications where the interest of the population is high, spectroscopy by smartphones is an open field for exploring new types of measurement campaigns, while also influencing and driving the environmental policies of the future.

## Figures and Tables

**Table 1:** Short codes identifying color samples.

Color Standards	Code	Color Fabrics	Code
Red	RD	Dark Red	DR
Green	GN	Dark Orange	OR
Blue	BL	Mustard	MU
Yellow	YW	Green	GR
Cyan	CY	Electric Blue	EB
Orange	OR	Dark Pink	DP
Purple	PU	Brick Red	BR
Violet	VI	Brown	BW
		Tortora	TO
		Blue	BL
		Black	BK
		Light Beige	LB

**Table 2:** Differences between certified and smartphone-based color measurements.

Tiles					Cloth Fabrics				
Code-Color	RGB		GoSpectro		Code-Color	RGB		GoSpectro	
	ΔE*	δ_E*_	ΔE*	δ_E*_		ΔE*	δ_E*_	ΔE*	δ_E*_
RD-Red	85	8.0	33	1.1	BK-Black	79	5.1	26	1.8
GN-Green	85	3.3	28	1.1	BL-Blue	75	5.7	38	0.5
BL-Blue	86	4.0	31	0.9	BR-Brick Red	77	5.4	43	1.5
YW-Yellow	71	3.2	10	1.0	BW-Brown	65	8.1	36	1.2
CY-Cyan	78	3.0	17	0.8	DP-Dark Pink	70	4.3	41	2.2
OR-Orange	69	6.3	26	0.5	DR-Dark Red	74	15.0	39	1.4
PU-Purple	94	3.6	39	1.5	EB-Electric Blue	68	8.8	46	1.2
VI-Violet	90	8.4	26	0.9	GR-Green	73	10.0	43	1.7
					LB-Light Blue	67	3.3	39	2.2
					MU-Mustard	62	42.0	56	0.8
					OR-Dark Orange	71	22.0	53	1.9
					TO-Tortora	73	5.3	42	1.9
**Mean**	**82.2**	**5.0**	**26.2**	**1.0**		**71.2**	**11.2**	**41.8**	**1.5**

## Data Availability

All data can be obtained from corresponding author at request.
